# Prolonged Amphetamine-Dextroamphetamine Use: An Unrecognized Cause of Cardiomyopathy

**DOI:** 10.7759/cureus.80553

**Published:** 2025-03-14

**Authors:** Bola Habeb, Nilgun Demirag, John Retzloff

**Affiliations:** 1 Internal Medicine, University of Florida College of Medicine/Ascension Sacred Heart, Pensacola, USA

**Keywords:** adhd treatment monitoring in adults, amphetamine-dextroamphetamine, amphetamine-induced cardiomyopathy, cardiac transplant, medication overuse, nonischemic cardiomyopathy, prescribed adderall, short term mechanical circulatory support

## Abstract

Adderall (amphetamine-dextroamphetamine), manufactured by Shire, Teva, and Impax Pharmaceuticals (Lexington, MA), is a commonly prescribed stimulant for attention-deficit/hyperactivity disorder (ADHD). It contains a combination of mixed amphetamine salts that enhance the activity of dopamine and norepinephrine in the brain. While generally well-tolerated, prolonged use has been associated with adverse cardiovascular effects, including cardiomyopathy. This condition, characterized by structural and functional abnormalities of the heart muscle, can manifest as left ventricular hypertrophy, dilation, or systolic dysfunction. Chronic amphetamine exposure may contribute to cardiotoxicity through mechanisms such as increased oxidative stress, sympathetic overactivity, and direct myocardial toxicity. We present a case report highlighting the progression of Adderall-induced cardiomyopathy, its clinical presentation, and diagnostic challenges. Given the increasing use of stimulant medications, early recognition of cardiovascular risks is essential to prevent irreversible cardiac remodeling and heart failure. Further research is needed to elucidate long-term outcomes and optimal management strategies for affected patients.

## Introduction

Attention-deficit/hyperactivity disorder (ADHD) is one of the most common neurodevelopmental disorders in children and can continue into adulthood. According to the American Psychiatric Association (APA), the prevalence of ADHD in the United States is estimated to be 8.4% in children and 2.5% in adults [1]. Typically, it is initially treated with behavioral therapy, which may be combined with stimulant or non-stimulant medications to help control behaviors that interfere with daily life activity. Adderall (Shire, Teva, and Impax Pharmaceuticals, Lexington, MA), one of the most frequently used stimulant medications, contains amphetamine salts, which include a combination of dextroamphetamine and levoamphetamine [2]. It has been prescribed for the therapeutic management of ADHD and narcolepsy. However, it is also frequently misused by adolescents and young adults without ADHD in an attempt to enhance academic performance, often without awareness of the potential risks [3].

Prolonged use of Adderall, particularly at high doses, can lead to significant cardiac side effects, including hypertension, tachycardia, arrhythmias, and an increased risk of myocardial infarction [4]. Chronic stimulant exposure stimulates excessive catecholamine release, which can lead to persistent vasoconstriction, increased myocardial oxygen demand, and structural cardiac changes. Over time, this may contribute to left ventricular hypertrophy, dilated cardiomyopathy, and heart failure [2,3].

Adderall-induced cardiomyopathy typically presents with symptoms such as progressive dyspnea, fatigue, palpitations, chest pain, and peripheral edema. Diagnosis requires a thorough clinical evaluation, including a detailed history of stimulant use, physical examination findings like tachycardia or volume overload, and advanced cardiac testing. An electrocardiogram (EKG) may reveal tachyarrhythmias or QT prolongation, while an echocardiogram can confirm reduced ejection fraction (EF) and ventricular dysfunction. Additional tests, such as cardiac MRI, serum biomarkers (beta natriuretic peptide (BNP), troponins), chest x-ray, and, if needed, cardiac catheterization, help rule out other causes of cardiomyopathy [2-4]. Early identification is essential, as discontinuing Adderall and initiating appropriate heart failure management can improve cardiac function and potentially reverse the condition in some cases.

Here, we discuss a case of a 40-year-old male patient with prolonged Adderall use for more than three decades who developed non-ischemic cardiomyopathy. His cardiac function deteriorated to the point of heart failure with reduced EF (HFrEF) (EF of 15%) and cardiogenic shock, necessitating temporary mechanical circulatory support placement and subsequent cardiac transplantation.

## Case presentation

A 40-year-old male with a medical history significant for ADHD and HFrEF diagnosed one year prior presented to our facility for evaluation of progressively worsening dyspnea, orthopnea, paroxysmal nocturnal dyspnea, abdominal distension, and bilateral lower extremity swellings, findings consistent with heart failure. At the time of his initial diagnosis, the patient was found to have a severely reduced EF of 10%-15% and subsequently experienced multiple hospitalizations due to acute-on-chronic systolic heart failure exacerbations. Following the diagnosis of HFrEF, guideline-directed medical therapy (GDMT), including sacubitril/valsartan, metoprolol succinate, dapagliflozin, spironolactone, and furosemide, was gradually initiated. The patient has remained adherent to the prescribed treatment regimen. Notably, alongside his heart failure medications, he has been taking 40 mg of oral Adderall daily, the maximum recommended dose, for ADHD management without interruption since the age of eight. He denied any tobacco, alcohol, or illicit drug use. He reported no family history of heart disease. 

Physical examination revealed a temperature of 36.5°C, blood pressure of 119/89 mmHg, pulse of 89 beats per minute (bpm), 18 respirations per minute, and oxygen saturation of 95% on ambient air. He appeared nontoxic and in no acute distress. The cardiovascular exam showed a regular rate and rhythm with an apical holosystolic murmur. Pulmonary examination demonstrated bilateral basal lung crackles. The abdomen was distended with mild diffuse tenderness. There was +2 bilateral lower extremity edema.

Admission laboratory results were unremarkable, except for an elevated BNP compared to the patient's baseline, with normal thyroid function and troponin levels (Table 1). The urine drug screen yielded a false positive for methamphetamine due to cross-reactivity with Adderall, while tests for cocaine, opiates, phencyclidine, methadone, cannabinoids, barbiturates, and benzodiazepines were all negative.

**Table 1:** Laboratory data on admission. AST: aspartate aminotransferase; ALT: alanine aminotransferase; BNP: beta natriuretic peptide; TSH: thyroid-stimulating hormone * Abnormal lab values.

Parameters	Patient’s values on admission	Reference range, adults
Hemoglobin (g/dL)	14	12.0–15.5
White cell count (per mm^3^)	7,800	3,500–10,500
Platelet count (per mm^3^)	328,000	150,000–450,000
Sodium (mEq/dL)	140	135–145
Potassium (mEq/dL)	4.7	3.5–5.1
Bicarbonate (mEq/dL)	22	22–29
Creatinine (mg/dL)	1.24	0.7–1.2
AST (units/L)	47	12–31
ALT (units/L)	47	9–29
Troponin (mg/mL)	0.012	0.010-0.033
BNP (pg/mL)	9,753.8*	10-100
Lactic acid (mmol/L)	1.8	0.5-2.2
TSH	2.48	0.35-4.94
Hemoglobin A1C	5.5	≤6.5

**Figure 1:**
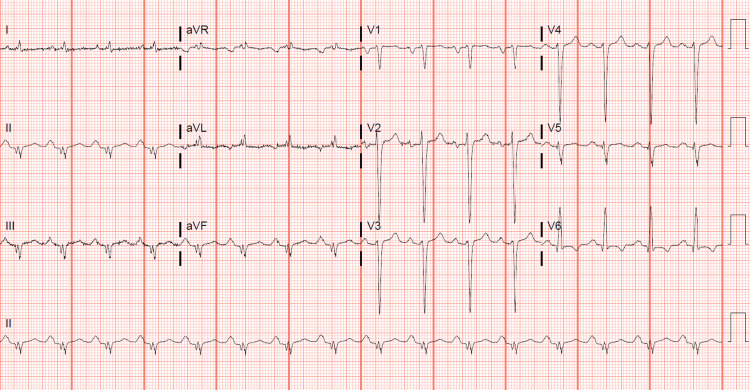
EKG demonstrating normal sinus rhythm, biatrial enlargement, and evidence of LVH. EKG: electrocardiogram; LVH: left ventricular hypertrophy

**Figure 2:**
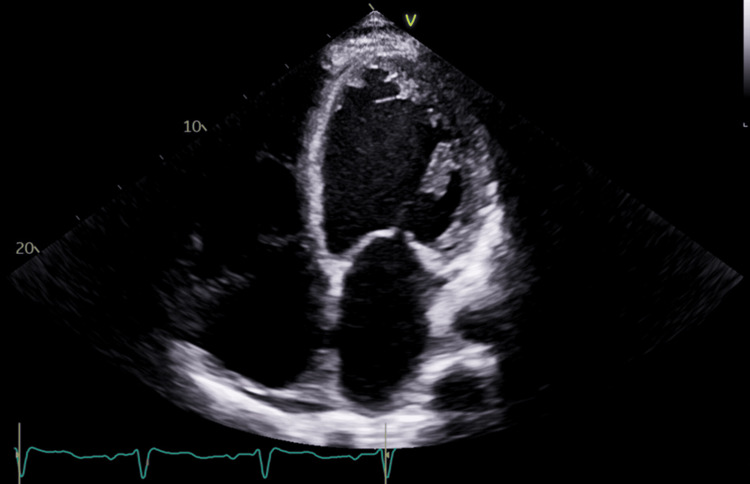
Apical four-chamber view echocardiogram showing biventricular enlargement with severely reduced systolic function (EF 15%). EF: ejection fraction

**Figure 3:**
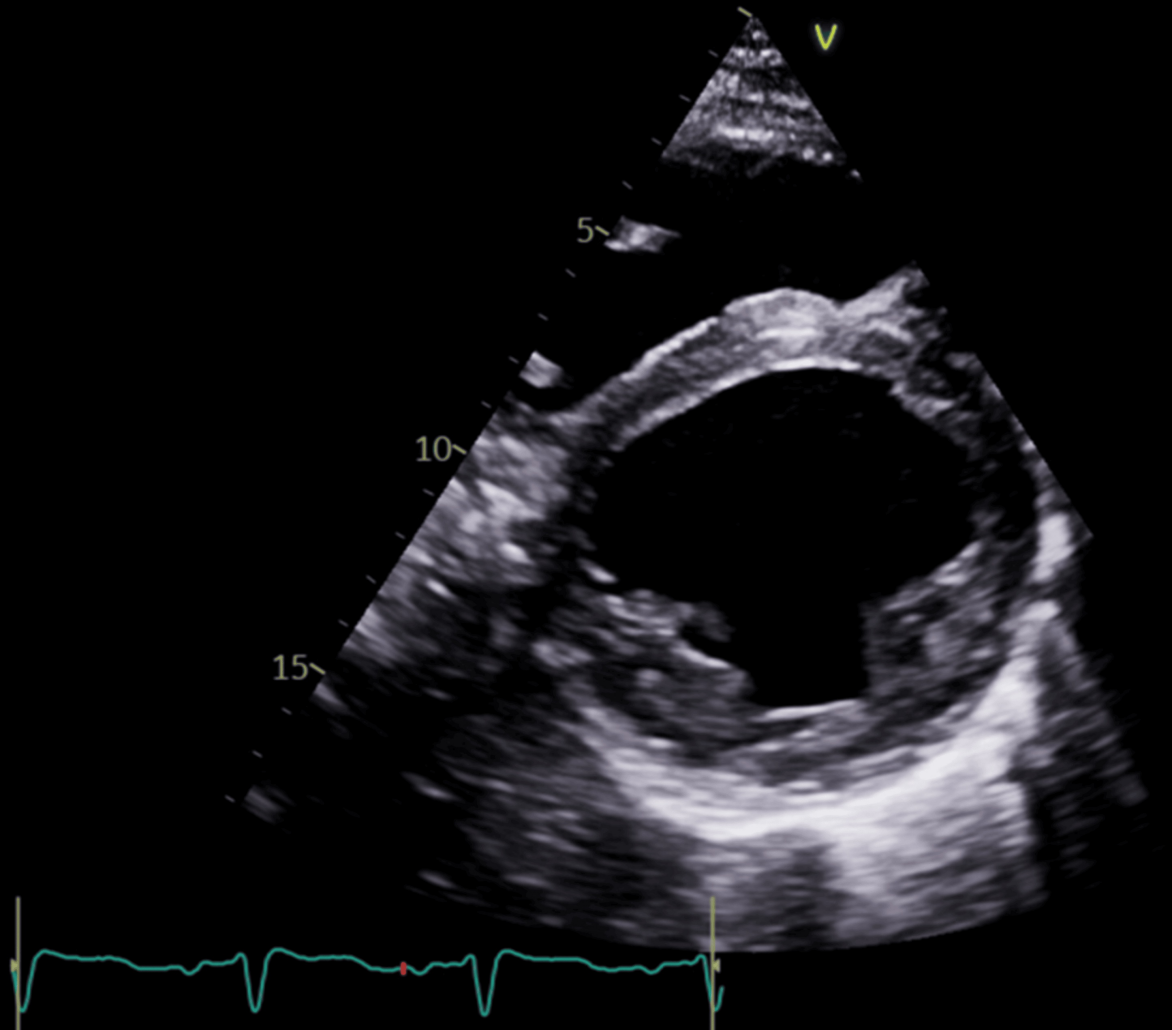
Parasternal short-axis view echocardiogram showing biventricular enlargement with reduced systolic function (EF 15%). EF: ejection fraction

His EKG revealed normal sinus rhythm with evidence of left ventricular hypertrophy (LVH) and no acute ST-segment abnormalities (Figure 1). The echocardiogram revealed a dilated left ventricle (LVEDV 6.97 cm) with severely reduced function, a calculated EF of 15%, and global hypokinesis. Additionally, there was a dilated right ventricle with moderately to severely reduced function, an estimated right ventricular systolic pressure (RVSP) of 45 mmHg, mild mitral regurgitation, and mild to moderate tricuspid regurgitation (Figures 2, 3).

Left heart catheterization revealed normal coronary vessels. Right heart catheterization showed a severely depressed cardiac index of 1 that was consistent with the Society for Cardiovascular Angiography And Interventions (SCAI) classification of cardiogenic shock stage B. Consequently, the patient was initiated on milrinone infusion, and an Impella device (Abiomed, Inc., Danvers, MA) was placed through a transfemoral approach at P5.5 for mechanical circulatory support. Since any meaningful LV reverse remodeling was unlikely given the chronicity of heart failure despite GDMT for the last six months, the patient was transferred to another facility for evaluation for a cardiac transplant.

## Discussion

ADHD is the most prevalent neurobehavioral disorder, affecting approximately 5% to 10% of school-aged children. While it was once believed to largely resolve during adolescence, emerging research increasingly indicates that the disorder and/or its associated impairments persist into adulthood in the majority of cases [4]. According to the Diagnostic and Statistical Manual of Mental Disorders (DSM-5), ADHD is characterized by persistent inattention and/or hyperactivity-impulsivity that significantly impacts daily functioning. Diagnosis requires at least six symptoms in children (or five in adolescents and adults) that persist for at least six months in a manner inappropriate for the developmental level. Symptoms of inattention include difficulty sustaining focus, forgetfulness, disorganization, and frequent distractions, while hyperactivity-impulsivity symptoms include excessive talking, restlessness, difficulty waiting turns, and impulsive interruptions. To confirm an ADHD diagnosis, symptoms must have been present before age 12, occur in multiple settings, impair daily functioning, and not be better explained by another condition [5].

Adderall is widely prescribed for ADHD and narcolepsy; however, its use, particularly at high doses or when misused by adolescents to boost wakefulness and academic performance, can lead to serious cardiovascular complications. A key concern is cardiomyopathy, with its risk significantly heightened by prolonged use, underlying heart conditions, or the concurrent use of other stimulants [4]. Amphetamine-induced catecholamine signaling plays a central role in the development of cardiomyopathy [6]. Persistent catecholamine exposure affects cardiac function through multiple mechanisms. First, increased vasoconstriction occurs due to elevated norepinephrine and dopamine levels, stimulating the central nervous system and leading to sustained increases in heart rate and blood pressure. Over time, this chronic elevation places excessive strain on the heart, potentially culminating in heart failure, particularly in individuals with pre-existing cardiovascular conditions. Additionally, Adderall-induced vasoconstriction contributes to an increased cardiac workload by raising afterload, subsequently leading to LVH and impaired contractility. Moreover, prolonged amphetamine use heightens the risk of arrhythmias and persistent tachycardia, which can progress to arrhythmia-mediated cardiomyopathy [7]. Furthermore, the drug's impact on vascular integrity may contribute to the development of coronary artery disease (CAD) by exacerbating hypertension and vascular damage, thereby reducing coronary blood flow [4]. Diminished myocardial perfusion can ultimately lead to ischemic cardiomyopathy and heart failure.

Previous studies assessing the safety of stimulant medications have predominantly focused on the first one to two years of use and found no evidence of short-term adverse cardiac effects [8]. Since many patients are prescribed these medications in early childhood and continue taking them into adulthood, a pooled analysis presented at the American Heart Association's (ACC) Scientific Sessions in 2024 examined 17 studies encompassing 433,392 patients with amphetamine misuse. The analysis revealed that 50.7% of these individuals had hypertension, 25.72% experienced ischemic heart disease, and 15.89% suffered myocardial infarctions [9].

Additionally, another study presented at the ACC’s Annual Scientific Session examined the impact of prolonged Adderall use on cardiomyopathy. Researchers found that young adults prescribed stimulant medications for ADHD were significantly more likely to develop cardiomyopathy compared to those not on these medications. Specifically, the risk was 17% higher after one year and 57% higher after eight years of stimulant use [8]. It is important to note, however, that the overall prevalence of amphetamine-induced cardiomyopathy remains relatively low, with the annual likelihood of occurrence being approximately one in 2,000 patients, though it rises to one in 500 for those using these medications for 10 years [8].

Notably, the patient denied alcohol consumption and illicit drug use, which ruled out toxic cardiomyopathy. He also reported no symptoms suggestive of a viral infection before the onset of heart failure, thereby excluding viral cardiomyopathy. Furthermore, a normal coronary angiogram ruled out ischemic cardiomyopathy, and the absence of a family history of heart disease helped exclude genetic heart conditions. The prolonged, consistent use of Adderall for more than three decades, in conjunction with the exclusion of other risk factors for cardiomyopathy, suggests prolonged stimulant medication use is most likely the etiology of this patient’s pathology.

The treatment strategy includes discontinuing Adderall, initiating GDMT for heart failure, lifestyle modifications (e.g., reducing salt intake, managing blood pressure, and avoiding stimulants), and advanced procedural interventions when indicated (e.g., primary prevention implantable cardioverter-defibrillator or heart transplant in severe irreversible cases) [10].

The outcome varies depending on the extent of heart damage and how early the condition is detected and treated. Some patients may recover partially or fully if Adderall is discontinued early, while others may experience permanent heart dysfunction [11,12].

## Conclusions

The widespread use of stimulant medications, particularly Adderall, underscores the importance of accurately diagnosing ADHD based on DSM-5 criteria. While these medications are highly effective in managing ADHD symptoms, they carry potential cardiovascular risks that, although rare, should not be overlooked. Adderall-induced cardiomyopathy is a serious, often underrecognized condition, especially in adolescents and young adults. Early detection and management are critical, as prompt intervention, including discontinuation of the stimulant and appropriate medical therapy, may improve cardiac function in some cases. However, delayed diagnosis or continued use of stimulants can lead to worsening heart failure and irreversible cardiac damage.

Given the increasing reports of Adderall-induced cardiomyopathy, a multidisciplinary approach involving primary care physicians, cardiologists, and mental health professionals is essential to carefully balance the benefits of ADHD treatment with the associated cardiovascular risks. Patients taking stimulant medications should be closely monitored for signs of heart failure, with echocardiograms recommended as needed. While the overall risk of cardiomyopathy remains low, the rising incidence of fatal cases emphasizes the need for cautious stimulant use. Further research is necessary to develop comprehensive guidelines for the safe use of stimulant medications, particularly regarding their long-term cardiovascular effects, and to establish effective screening tools for at-risk patient populations.
